# Minimal Influence of Hypobaria on Heart Rate Variability in Hypoxia and Normoxia

**DOI:** 10.3389/fphys.2020.01072

**Published:** 2020-08-21

**Authors:** Mathias Roland Aebi, Nicolas Bourdillon, Denis Bron, Grégoire P. Millet

**Affiliations:** ^1^Swiss Aeromedical Center, Swiss Air Force, Dübendorf, Switzerland; ^2^Institute of Sport Sciences, University of Lausanne, Lausanne, Switzerland; ^3^Armasuisse, Wissenschaft and Technologie, Thun, Switzerland; ^4^Be.care SA, Renens, Switzerland

**Keywords:** normobaric normoxia, normobaric hypoxia, hypobaric normoxia, hypobaric hypoxia, heart rate variability

## Abstract

**Introduction:**

The present study evaluated the putative effect of hypobaria on resting HRV in normoxia and hypoxia.

**Methods:**

Fifteen young pilot trainees were exposed to five different conditions in a randomized order: normobaric normoxia (NN, P_B_ = 726 ± 5 mmHg, F_I_O_2_ = 20.9%), hypobaric normoxia (HN, P_B_ = 380 ± 6 mmHg, F_I_O_2_≅40%), normobaric hypoxia (NH, P_B_ = 725 ± 4 mmHg, F_I_O_2_≅11%); and hypobaric hypoxia (HH at 3000 and 5500 m, HH3000 and HH5500, P_B_ = 525 ± 6 and 380 ± 8 mmHg, respectively, F_I_O_2_ = 20.9%). HRV and pulse arterial oxygen saturation (SpO_2_) were measured at rest seated during a 6 min period in each condition. HRV parameters were analyzed (Kubios HVR Standard, V 3.0) for time (RMSSD) and frequency (LF, HF, LF/HF ratio, and total power). Gas exchanges were collected at rest for 10 min following HRV recording.

**Results:**

SpO_2_ decreased in HH3000 (95 ± 3) and HH5500 (81 ± 5), when compared to NN (99 ± 0). SpO_2_ was higher in NH (86 ± 4) than HH5500 but similar between HN (98 ± 2) and NN. Participants showed lower RMSSD and total power values in NH and HH5500 when compared to NN. In hypoxia, LF/HF ratio was greater in HH5500 than NH, whereas in normoxia, LF/HF ratio was lower in HN than NN. Minute ventilation was higher in HH5500 than in all other conditions.

**Discussion:**

The present study reports a slight hypobaric effect either in normoxia or in hypoxia on some HRV parameters. In hypoxia, with a more prominent sympathetic activation, the hypobaric effect is likely due to the greater ventilation stimulus and larger desaturation. In normoxia, the HRV differences may come from the hyperoxic breathing and slight breathing pattern change due to hypobaria in HN.

## Introduction

Environmental hypoxia is a condition characterized by a decrease in the inspired oxygen pressure (P_I_O_2_) (Millet et al., 2012), which *per se* has a negative influence on autonomic cardiac response (Botek et al., 2015) and induces systemic/integrative metabolic, endocrine and vascular compensation (Marshall, 1998). More precisely, acute hypoxic exposure induces decreases in heart rate variability (HRV) and parasympathetic activity (Wille et al., 2012), whereas sympathetic activity increases (Richalet et al., 1988; Marshall, 1994). Contrastingly, HRV parameters 24 h after maximal anaerobic exercise comparing normoxic with two normobaric hypoxic conditions (equivalent to 2500 and 4000 m) remained unchanged (Álvarez-Herms et al., 2020). Therefore, the duration of hypoxic exposures as well as the timing of the HRV measurement probably influence HRV modulation. However, it is well established that high-altitude leads to sympathetic activation (Hainsworth et al., 2007), also under the influence of the rate of ascent (Vogel and Harris, 1967).

Heart rate variability is a non-invasive method to assess the cardiac autonomic control (Buchheit, 2014) and is commonly used to monitor fatigue and overreaching in athletes (Meeusen et al., 2013; Bourdillon et al., 2017), despite some debates about the pro and cons of the time (i.e., root mean square of the successive differences, RMSSD) (Plews et al., 2012) vs. frequency [i.e., spectral power in low frequency (LF), high frequency (HF) and total power (LF + HF)] domain HRV parameters (Schmitt et al., 2015).

Environmental hypoxia can be provoked either by lowering inspired oxygen fraction (F_I_O_2_; normobaric hypoxia, NH) or using a hypobaric chamber by reducing the barometric pressure (P_B_; hypobaric hypoxia, HH). For long, it was believed that all responses to hypoxia were only caused by the alveolar oxygen pressure (P_A_O_2_) decrease (Conkin, 2016). Contradictory, HH is suggested as a more severe environmental condition than NH (Millet et al., 2012). Several differences between NH and HH were reported, such as minute ventilation (Savourey et al., 2003), oxydative stress (Faiss et al., 2013), sleep disturbance (Saugy et al., 2016), and cerebrovascular function (Aebi et al., 2020). Therefore, NH and HH are not interchangeable (Conkin, 2016) but the clinical significance of the difference remains highly debated (Millet and Debevec, 2020; Richalet, 2020).

Isolating the hypobaric effect from the hypoxic one would allow comparing similar normoxic conditions with different P_B_. A hypobaric normoxic (HN) condition (i.e., low P_B_ and hyperoxic breathing in order to obtain a comparable P_I_O_2_ than in normobaric normoxia, NN) is therefore of interest for evaluating the putative hypobaric effect in normoxia (Millet and Debevec, 2020). Moreover, there is a practical interest of the present study since hypobaric normoxia occurs in the context of aviation; i.e., for pilots exposed to hypobaria in cockpit using supplemental oxygen. More precisely, pilots during flights at high-altitude may be exposed to hypobaria in unpressurized cabin aircraft, in case of sudden cabin depressurization during commercial flights or in military aircraft while breathing hyperoxic gas mixture. Hypobaric normoxia is also used for workers (i.e., miners in Chile) exposed to high terrestrial altitude with supplemental oxygen for example in dormitories for reducing periodic breathing and improving recovery (Moraga et al., 2014). Due to lower air density, a recent study showed ventilatory pattern change (i.e., increased maximal ventilation) in such environment (Ogawa et al., 2019). Moreover, increase in intrapulmonary pressure has been reported (Conkin, 2016). These physiological changes may impact HRV parameters, as the cardiac autonomic activity is influenced by the respiration (Brown et al., 1993) and the pulmonary arterial baroreceptors (Hainsworth et al., 2007). It was also suggested that parasympathetic influence increases in HN (Prabhakaran and Tripathi, 2011).

The present study evaluated first the altitude level influence on HRV during acute HH exposure at 3000 and 5500 m when compared to NN. More importantly, we investigated the putative effect of hypobaria on HRV during acute exposure in hypoxia (NH vs. HH) and in normoxia (NN vs. HN).

## Materials and Methods

### Participants and Protocol Design

Fifteen healthy pilot trainees (26 ± 4 years, 177 ± 7 cm, 71 ± 9 kg) were exposed to five different conditions in a randomized order: NN (440 m, P_B_ = 726 ± 5 mmHg, F_I_O_2_ = 20.9%); NH (simulated altitude of 5500 m, P_B_ = 725 ± 4 mmHg, F_I_O_2_≅11%); HN (depressurization at 5500 m with hyperoxic breathing to avoid hypoxia in hypobaria, P_B_ = 380 ± 6 mmHg, F_I_O_2_≅40%) and HH (P_B_ = 525 ± 6 and 380 ± 8 mmHg, for 3000 m (HH3000) and 5500 m (HH5500) respectively, F_I_O_2_ = 20.9%). Gas mixtures employed for NH and HN conditions were prefilled in cylinders. Participants breathed 100% of oxygen during altitude elevation (i.e., during atmospheric pressure reduction in the hypobaric chamber). Decompression lasts for around 2 min in the hypobaric conditions (HH and HN). A physician screened the participants during a familiarization visit to ensure they were healthy and did not report any medical or altitude related issues.

Twenty-four hours before test visit, participants were asked to avoid physical exercise and consuming a heavy meal, alcohol and caffeine. Participants remained at rest, seated, during the entire experimental procedures. Each tested condition consisted 5 min of condition acclimatization followed by 6 min seated at rest. Then, participants also performed a concentration test [arithmetic tasks including working memory, KLT-R test (Düker and Lienert, 2001)] and hypercapnic breathing protocol to assess cerebrovascular reactivity to CO_2_ (Aebi et al., 2020). Each period lasted for 30 min, interspaced by a 30 min rest period in NN, for total session duration of 5 h.

In order to evaluate putative hypobaric effect between normoxic and hypoxic conditions with comparable P_I_O_2_: NN vs. HN (141 ± 1 vs. 133 ± 3 mmHg) and NH vs. HH5500 (74 ± 1 vs. 70 ± 2 mmHg) were compared by adjusting P_B_ in the hypobaric chamber or F_I_O_2_ (i.e., ≈11% and ≈40% O_2_ gas mixture for NH and HN, respectively) based on known equation (P_I_O_2_ = (P_B_-47) × F_I_O_2_), when the water vapor pressure at 37°C is 47 mmHg (Conkin, 2016).

### Measurements

Heart rate variability was recorded with heart rate monitor (Polar RS800CX, FI-90440 Kempele, Finland). HRV measurement was performed according to previous findings of our research group (Bourdillon et al., 2017), during the last 4 min of a 6 min rest period seated (i.e., around 300 beats were analyzed). HRV data were analyzed using specific software (Kubios HVR Standard, V 3.0). Time domain HRV index (RMSSD) and spectral power for frequency bands for: HF (0.15–0.50 Hz), LF (0.04–0.15 Hz) and total power (LF + HF) were analyzed. LF/HF ratio was calculated to evaluate the sympathovagal balance.

Pulse oxygen saturation (SpO_2_,%) was monitored at the left earlobe using an oximeter (3100 pulse oximeter, Nonin, Plymouth, MN) and acquired at 0.5 Hz. Mean SpO_2_ was calculated during the last minute of rest period in each condition.

Gas exchanges data were recorded using a gas analyzer (K5, Cosmed, Roma, Italy) that was calibrated outside of the hypobaric chamber before each session. Flow volume was calibrated with a 3L syringe. After calibrating zero CO_2_ with scrubber, reference gas was assessed using a certified Cosmed gas concentration (16% O_2_ and 5% CO_2_). Ventilatory data were recorded by the analyzer and exported in Cosmed software for later analysis (OMNIA, Cosmed, Roma, Italy).

### Statistical Analysis

Repeated measures ANOVA were assessed for condition comparison for absolute values. Greenhouse-Geisser sphericity correction was applied when Mauchly’s test statistic was significant (*p* < 0.05). Then, Tukey *Post hoc* test was performed for condition comparison. Statistical analysis was performed separately for altitude comparison (NN, HH3000 and HH5500) and for conditions comparison (NN, HN, NH, and HH5500). Repeated measures ANOVA (non-parametric, Friedman) were performed for relative (%Δ) changes from NN values. Statistical analysis was assessed using Jamovi software (Jamovi project 2018, version 0.9). Significant difference was set for *p* < 0.05.

### Ethical Approval

This study was performed according to the Declaration of Helsinki and was approved by the Swiss Ethic Committee of Zürich (Swissethics, BASEC ID: 2017-00752). This clinical trial can be found on ClinicalTrials.gov (ID: NCT03303118). All participants were informed about all procedures of this study and gave their written informed consent before participating to this study.

## Results and Discussion

### Altitude Level Influence in Hypobaric Hypoxia

All absolute physiological data for HH conditions are displayed in Table 1. As expected, HR gradually increased with altitude level in HH3000 (*p* = 0.014) and HH5500 (*p* < 0.001) when compared to NN. RMSSD decreased in HH3000 (*p* = 0.013) and HH5500 (*p* < 0.001) when compared to NN. LF and total power absolute values were lower in HH5500 and HH3000 than in NN. Moreover, relative changes in LF and total power were greater in HH5500 vs. HH3000 (-59 vs. -41%, *p* = 0.047 and -61 vs. -44%, *p* = 0.047, for LF and total power, respectively). Previously, decrease in total power was also observed at high altitude (Hughson et al., 1994; Sevre et al., 2001), in line with the present results. Moreover, total power reduction indicates a reduced autonomic heart rate control (Kautzner and John Camm, 1997). Despite a significant HR increase, HF (ms^2^) did not significantly decrease in HH3000 (*p* = 0.17). However, HF (ms^2^) significantly decreased in HH5500 (*p* = 0.004), when compared to NN. Several studies have suggested a shift in the balance of the autonomic nervous system toward relatively less parasympathetic and more sympathetic activity at high altitude (Hughson et al., 1994; Perini et al., 1996; Sevre et al., 2001). Overall, HRV and parasympathetic activity decreased (i.e., RMSSD, HF, LF and total power reduction) with altitude elevation in acute HH, therefore to greater extent at 5500 m. The present results suggest a larger predominance of the sympathetic activity in hypobaric hypoxia.

**TABLE 1:** Absolute values are means ± *SD*.

	NN	HH3000	HH5500
HR (bpm)	73.4 ± 7.0	81.4 ± 10.2*	93.0 ± 14.2***§§§
RMSSD (ms)	46.1 ± 15.6	37.5 ± 20.1*	25.5 ± 16.1***
LF (ms^2^)	2381 ± 1311	1405 ± 1162**	783 ± 536***
HF (ms^2^)	816 ± 442	606 ± 531	311 ± 314**
LF (n.u.)	73.9 ± 9.4	72.3 ± 13.5	75.1 ± 14.1
HF (n.u.)	25.8 ± 9.5	25.5 ± 10.7	24.9 ± 14.0
LF/HF ratio	3.3 ± 1.2	3.4 ± 1.8	4.4 ± 2.9
Total Power (LF + HF)	3197 ± 1629	1703 ± 112**	999 ± 602***
SpO_2_ (%)	99.5 ± 0.4	95.4 ± 2.7**	81.3 ± 5.5***§§§

Physiological variables (n = 15): HR, Heart rate; RMSSD, root mean square of the successive differences; LF, low frequency; HF, high frequency; SpO_2_, pulse oxygen saturation in NN, normobaric normoxia in hypobaric hypoxia at 3000 m and 5500 m (HH3000 and HH5500). * p < 0.05, ** p < 0.01, *** p < 0.001 for difference with NN. ^§^p < 0.05, ^§§§^p < 0.001 for difference with HH3000.

### Slight Additional Effect of Hypobaria in Both Hypoxia and Normoxia

**FIGURE 1:**
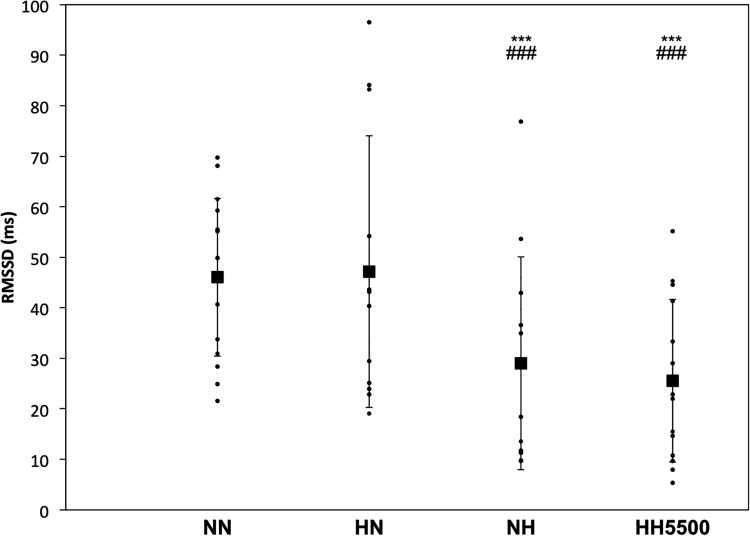
The root mean square of the successive differences (RMSSD) for each subject. Bold squares represent absolute means ± *SD* for conditions: NN, Normobaric normoxia; HN, hypobaric normoxia; NH, normobaric hypoxia; HH5500, hypobaric hypoxia at 5500 m. ^∗∗∗^*p* < 0.001 for difference with NN. ^###^*p* < 0.001 for difference with HN.

HR increased in both NH and HH5500 when compared to NN and HN (*p* < 0.001), but to larger extent in HH5500 than NH (Table 2), which confirms previous findings (Savourey et al., 2003; Self et al., 2011). However, RMSSD decreased in NH and HH5500 likewise in comparison to NN and HN (*p* < 0.001, Figure 1). Moreover, LF (ms^2^) was lower in NH and HH5500 (*p* < 0.01 and *p* < 0.001, respectively) than NN. HF (ms^2^) was lower in HH5500 than in HN (*p* = 0.025). More precisely, decreases in HF were greater in NH (-35%, *p* = 0.048) and HH5500 (-60%, *p* < 0.001) than in HN (+ 8%), when compared to NN. Moreover, reduction in HF was also larger in HH5500 than in NH (*p* = 0.048), which implies a greater parasympathetic activity reduction. Total power decreased in NH (*p* = 0.035) and HH5500 (*p* = 0.004) when compared to HN and NN (*p* < 0.001). Sevre et al. (2001) demonstrated a transient reduction in parasympathetic and sympathetic activity (i.e., decreased total power, LF and HF power) during stepwise exposure to high altitude. The present results confirm previous findings suggesting HRV reduction (Wille et al., 2012), sympathetic activity elevation (Richalet et al., 1989; Marshall, 1994), and sympathetic predominance during acute exposure to hypoxia (Chen et al., 2008; Wille et al., 2012).

**TABLE 2:** Absolute values are means ± *SD.*

	Normoxia		Hypoxia	
	NN	HN	NH	HH5500
HR (bpm)	73.4 ± 7.0	77.4 ± 10.7	86.9 ± 13.2*** ###	93.0 ± 14.2*** ###^††^
RMSSD (ms)	46.1 ± 15.6	47.1 ± 26.9	29.0 ± 21.1*** ###	25.5 ± 16.1*** ###
LF (ms^2^)	2381 ± 1311	1908 ± 1833	1176 ± 1178***	783 ± 536*** ##
HF (ms^2^)	816 ± 442	987 ± 1058	661 ± 885	311 ± 314#
LF (n.u.)	73.9 ± 9.4	61.0 ± 12.7	68.8 ± 10.4	75.1 ± 14.1
HF (n.u.)	25.8 ± 9.5	39.0 ± 12.7	31.2 ± 10.4	24.9 ± 14.0
LF/HF ratio	3.3 ± 1.2	1.6 ± 0.9*	2.6 ± 1.3	4.4 ± 2.9###†
Total Power (LF + HF)	3197 ± 1627	2895 ± 2719	1433 ± 1466** #	999 ± 602*** ##
SpO_2_(%)	99.5 ± 0.4	98.4 ± 1.8	86.0 ± 4.5*** ###	81.3 ± 5.5*** ###^††^

Physiological variables (n = 15). HR, Heart rate; RMSSD, root mean square of the successive differences; LF, low frequency; HF, high frequency and SpO_2_, pulse oxygen saturation in each condition: NN, Normobaric normoxia; HN, hypobaric normoxia; NH, normobaric hypoxia; HH5500, hypobaric hypoxia at 5500 m. * p < 0.05, ** p = 0.002, *** p < 0.001 for difference with NN. ^#^p < 0.05, ^##^p < 0.01, ^###^p < 0.001 for difference with HN. ^†^p < 0.05, ^††^p < 0.01 for difference with NH.

Acute hypoxia is considered as a potent activator of sympathetic activity (Richalet et al., 1988; Marshall, 1994; Hainsworth et al., 2007). When exposed to acute hypoxia, the muscle sympathetic nerve activity (MSNA) increases (Duplain et al., 1999; Hansen and Sander, 2003), due to the hypoxia-induced sympathetic activation (Marshall, 1994). LF/HF ratio was higher in HH5500 than HN (*p* < 0.001), which confirms the hypoxia-induced sympathetic activity elevation (i.e., with similar barometric pressure between HN and HH5500). Interestingly, LF/HF was greater in HH5500 than NH, which may imply a slight hypobaric additional influence on sympathetic activation commonly reported in hypoxia. However, since there was no other significant difference in HRV parameters between HH5500 and NH, we assume that the present experimental evidences are not strong enough for such statement about the influence of hypobaria on HRV in hypoxia.

In normoxic conditions, HR was similar between NN and HN. Nevertheless, some differences were found between NN and HN for some HRV indices, suggesting a slight hypobaric influence on HRV in normoxia at rest: LF/HF ratio was lower in HN than NN (*p* = 0.041), suggesting parasympathetic activity predominance in HN. However, HF was similar and LF did not significantly decrease in HN (*p* = 0.105) when compared to NN. Parasympathetic increase was observed in subjects exposed to 4574 m breathing enriched O_2_ gas mixture (Prabhakaran and Tripathi, 2011). This may be related with a decreased MSNA when breathing a hyperoxic gas mixture (Querido et al., 2010). In fact, peripheral chemoreceptors seem inhibited with hyperoxic stimulus leading to MSNA reduction (Querido et al., 2010). Moreover, change in breathing pattern due to lower air density in hypobaria, may be an additional factor to take into account (Ogawa et al., 2019). Despite non-significant difference, our data pointed this breathing pattern change in hypobaria, with lower ventilation value in HN than NN.

### Influence of Ventilation on Heart Rate Variability

It is known that the cardiac autonomic nerve activity is influenced by ventilation (Brown et al., 1993). In a parallel article from our laboratory, minute ventilation and breathing frequency significantly increased in HH5500, but not NH, when compared to NN at rest (Aebi et al., 2020). Moreover, tidal volume tended to be higher in HH5500 than NN, while it remained unchanged in NH (Aebi et al., 2020). Gas exchanges data were collected on nine of the fifteen participants for measuring the hypercapnic response to CO_2_. These data were collected 10 min following HRV measurement (Table 3). In fact, they should be interpreted with cautious, as it may not reflect accurately the gas exchanges during HRV recording, but it gives us insights of the ventilatory responses in each condition. We did not record ventilation and HRV during the same time period on purpose since wearing the mask may be a cofounding factor for resting HRV. The severity of the respiratory hypoxic response probably influenced HRV modulations. Therefore, HH5500 may induce slight greater cardiovascular stress than NH, possibly due to higher respiratory stimulus in addition to the hypoxic stimulus (i.e., greater minute ventilation). In the context of hypoxia, respiratory “stress” (i.e., hyperventilation in HH in the present study) may activate directly sensors and regulators of other integrative systems as endocrine-metabolic and cardiovascular. More precisely, gene expression and their immediate and long-term expression may also individually influence HRV. Hundreds of genes targeted in the HIF1-α pathway are potential candidates but it is beyond the scope and the results of the present study to further speculate on it.

**TABLE 3:** Absolute values are means ± *SD* (*n* = 9).

	NN	HH3000	HN	NH	HH5500
V_*E*_ (L/min)	12.1 ± 1.4	12.5 ± 1.4	10.3 ± 1.4	12.1 ± 2.7	16.0 ± 2.7^∗∗∗^^§^^ ###^^††^
BF (cycle/min)	15.9 ± 2.6	16.7 ± 2.8^∗∗^	17.9 ± 3.0	17.0 ± 3.6	17.9 ± 2.7^∗∗∗^
VT (L)	0.82 ± 0.21	0.79 ± 0.20	0.62 ± 0.21	0.79 ± 0.27	0.98 ± 0.31^∗^^§^^ ##^

Ventilatory parameters: Minute ventilation (VE), breathing frequency (BF) and tidal volume (VT) at rest. In normobaric normoxia (NN, Dübendorf altitude level of 440 m), hypobaric hypoxia (HH, at altitude level of 3000 m and 5500 m, HH3000 and HH5500), hypobaric normoxia (HN, altitude level of 5500 m in normoxia), and normobaric hypoxia (NH, altitude simulation of 5500 m in normobaria). Statistical analysis was performed separately for altitude comparison in HH (NN, HH3000 and HH5500) and for conditions comparison (NN, HN, NH, and HH5500). * p = 0.061, ** p < 0.01, and *** p < 0.001 different from NN. ^§^ p < 0.05 different from HH3000. ^#^p < 0.05, ^##^p < 0.01, and ^###^p < 0.001 different from HN. ^†^p < 0.05 different from NH.

HRV analysis requires caution, as it may not reflect pure autonomic tone and is influenced by several regulation loops as baroreflex or respiratory sinus arrhythmia. In addition, interpretation in LF/HF is popular but controversial (Williams et al., unpublished). HF component of the power spectral analysis of HRV is affected by the respiratory modulation of the vagal nerve activity. LF component and how it relates to the sympathetic tone and the baroreflex remains controversial (Williams et al., unpublished). HRV spectral analysis appears more sensitive and helpful than time-domain HRV indices (Schmitt et al., 2015). As frequency spectral power parameters did not show significant changes, we believe that supplemental time-domain parameters (i.e., pNN50 and SDNN) would not add value to the present manuscript.

Overall, HRV decreased in hypoxic conditions, which is in line with a previous elegant study that showed a decrease in spectral components of heart rate variability (i.e., total power, LF and HF) when exercising in acute hypoxia (F_*I*_O_2_ = 11.5%) in comparison with exercise in normoxia (Povea et al., 2005). The present study adds novelty by suggesting a slight influence of hypobaria in both hypoxia and normoxia on HRV modulations through ventilation pattern differences. The alveolar air equation shows that the coupled alveolar O_2_ (P_*A*_O_2_) and carbon dioxide partial pressures (P_*A*_CO_2_) for NH and HH are not identical when P_*I*_O_2_ is equivalent (Fenn et al., 1946; Rahn and Otis, 1949; Rahn and Fenn, 1962). Therefore, physiological responses to NH cannot be identical to the responses to HH given only equivalent hypoxic P_*I*_O_2_. An integrated mechanism should start with the alveolar air equation, especially the contribution of N_2_ in setting the coupled P_*A*_O_2_ and P_*A*_CO_2_ partial pressures (Conkin, 2016).

### Heart Rate Variability and Its Potential Relation With Hypoxemia

As expected, SpO_2_ decreased in HH3000 (*p* = 0.003) and HH5500 (*p* < 0.001) when compared to NN, but to a greater extent in HH5500 (Table 1.). SpO_2_ was higher in normoxic conditions (NN and HN, *p* < 0.001) than in NH and HH5500 (Table 2). Moreover, SpO_2_ was lower in HH5500 than in NH (*p* = 0.002), which confirmed the greater hypoxemia induced by HH, when compared to NH, in line with several previous studies (Savourey et al., 2003; Saugy et al., 2016). It was previously shown that ΔSpO_2_ interacts with ΔLF/HF ratio (Botek et al., 2015). Moreover, Δ SpO_2_ was correlated with delta RMSSD using natural logarithm transformation (ΔLn RMSSD) during first 5 min of NH exposure (Krejèí et al., 2018). In the present study, %Δ HR was negatively correlated with%Δ SpO_2_ (*r* = -0.594, *p* = 0.046) in HH5500. Last but not least, %Δ SpO_2_ was positively correlated (*r* = 0.629, *p* = 0.032) with total power in HH5500 only. Therefore, the present results confirm potential relation between HRV modulations and SpO_2_ in acute hypoxia (i.e., during the first 10 min of exposure). In addition, time-dose may also play an important role in individual hypoxemic state and HRV modulation. Duration of flights often differ depending on the mission, which may influence stress perceived and tolerated by the pilot. A recent study demonstrated that hypoxic stimulus may improve the tolerance to discomfort in athletes during high –intensity exercise (Álvarez-Herms et al., 2016). It would thus be of interest to investigate how hypoxia and hypobaria would modulate HRV during different exposure durations.

In conclusion, the present study reports a slight hypobaric effect either in normoxia or in hypoxia. In normoxia, this effect is related to an increase of parasympathetic activation, likely due to the hyperoxic breathing in HN. In hypoxia, where hypobaria induced a more prominent sympathetic activation, the hypobaric effect is likely due to the greater ventilation stimulus and larger desaturation in HH5500 than in NH.

## Data Availability Statement

All datasets presented in this study are included in the article/supplementary material.

## Ethics Statement

The studies involving human participants were reviewed and approved by the Swiss Ethic Committee of Zürich, BASEC ID: 2017-00752. The patients/participants provided their written informed consent to participate in this study.

## Author Contributions

MA, NB, DB, and GM were part of the conception of the protocol. MA conducted the experiments, was responsible for data acquisition, and wrote the manuscript. MA conducted the analysis and interpreted the results with NB and GM. GM revised the manuscript critically and gave advices to MA for corrections. All authors reviewed and approved the manuscript prior to submission.

## Conflict of Interest

MA and NB were employed by the companies Armasuisse and Be.care, respectively. The remaining authors declare that the research was conducted in the absence of any commercial or financial relationships that could be construed as a potential conflict of interest.
